# Early Implementation of a Regional Telehealth Contingency Staffing Program and Primary Care Quality in the Veterans Health Administration: Evidence from the Clinical Resource Hub program

**DOI:** 10.1007/s11606-025-09615-2

**Published:** 2025-05-20

**Authors:** Chelle L. Wheat, Sara E. Kath, Karin M. Nelson, Idamay Curtis, Ashok Reddy

**Affiliations:** 1 Center for Veteran-Centered and Value-Driven Care, VA Puget Sound Health Care System, Seattle, WA USA; 2 Department of Health Systems and Population Health, University of Washington, Seattle, WA USA; 3 Division of General Internal Medicine, Department of Medicine, University of Washington, Seattle, WA USA; 4 Present Address: Primary Care Analytics Team, Veterans Affairs Puget Sound Healthcare System, Seattle, WA USA

**Keywords:** telehealth, contingency staffing, primary care quality, veterans

## Abstract

**Background:**

The Veterans Health Administration (VHA) launched the Clinical Resource Hub (CRH) program to address primary care staffing deficits and improve access.

**Objective:**

To determine if the quality of primary care at clinics that use CRH services was similar to that of clinics that did not. Secondarily, to examine this association for clinics that serve a high proportion of minority Veterans.

**Design:**

A quasi-experimental study using VHA administrative data from October 2017 through September 2021. We applied interrupted time series models to estimate changes in primary care quality measures associated with CRH utilization. Results are reported as percentages.

**Participants:**

National cohort of 107 propensity-matched VHA clinics that did and did not use CRH primary care services

**Intervention(s):**

CRH primary care services

**Main Measure(s):**

Chronic disease quality measures

**Key Results:**

For diabetes quality measures, we found similar results between CRH-utilizing clinics and their controls, including annual HbA1c screening (0.0% percentage difference (−1.0%, 1.0%), *p* = 0.640), poor HbA1c control (−1.0% (−1.0%, 0.0%), *p*=0.111), control of blood pressure for Veterans with diabetes (1.0% (−0.0%, 3.0%), *p*=0.095), statin therapy for Veterans with diabetes (1.0% (0.0%, 1.0%), *p*=0.003), statin adherence for Veterans with diabetes (0.0% (−1.0%, 0.0%), *p*=0.292), and nephropathy screening (1.0% (0.0%, 1.0%), *p*=0.010). There were no differences between clinic groups for control of blood pressure (1.0% (−1.0%, 2.0%),* p*=0.382). For cardiovascular quality measures, including statin therapy for Veterans with cardiovascular disease (0.0% (−1.0%, 2.0%), *p*=0.348), and statin adherence for Veterans with cardiovascular disease (−1.0% (−3.0%, 1.0%), *p*=0.467), we found no differences between clinic groups. Similar results were found among clinics that serve a high proportion of minority Veterans.

**Conclusions:**

We found that quality measures at CRH-utilizing clinics are similar to matched comparator clinics. These findings demonstrate that telehealth interventions, like CRH, can improve access to primary health care in a variety of settings, without impacting the quality of primary care.

**Supplementary Information:**

The online version contains supplementary material available at 10.1007/s11606-025-09615-2.

## INTRODUCTION

The Veterans Health Administration (VHA) has an increasing shortage of primary care providers, similar to other US healthcare systems^1,2^. These staffing shortages lead to reductions in patient access to care, which in turn can lead to disruptions in essential preventive and chronic disease services^3^, as well as an increase in mortality^4^. Physician shortages are worse in rural areas, with the workforce of primary care physicians decreasing across greater than half of rural US counties in recent years^5^. A large proportion of Veterans live in rural areas, so this is of significant concern to VHA^6^.

The Clinical Resource Hub (CRH) program was designed to address primary care staffing deficits and improve Veteran health care access to primary care, especially in medically underserved and rural areas^7,8^. When a VHA clinic site loses staff or experiences an unpredicted gap, the CRH program can provide support for patients at these sites. CRH uses a hub and spoke care model in which each regional hub is staffed with primary care providers and support staff that are deployed to spoke clinics. Clinical support is most often through telehealth modalities including telephone, video to clinic, and video to home. Decisions regarding how and when to deploy the CRH hubs are made at the regional level.

The evidence to date on whether large-scale telehealth interventions can provide care that is of equal quality to face-to-face care is mixed. A number of studies have found that while telehealth is practical and effective for certain types of care, it is less useful for care that requires a physical examination^9–11^. Moreover, telehealth may lead to more low-quality care. For example, the use of telehealth for acute respiratory visits has led to more unnecessary antibiotic prescriptions^12^. Another study found that hospitalizations for ambulatory-sensitive conditions, which are often used as an indicator for primary care access and quality, were higher among practices that used a high volume of telehealth compared to those that used a low volume^13^.

In addition to quality, another concern is that telehealth interventions may exacerbate existing disparities. The majority of studies have shown that older patients are less likely to engage with telemedicine services, as well as patients who are non-native English speakers or who reside in lower socioeconomic status areas^14^. Further, patients who reside in rural areas, or identify as a minority race, are less likely to use telehealth services^15–17^.

The national implementation of the CRH program provides a unique opportunity to address knowledge gaps in these key areas. The specific focus of the CRH program is to provide access to healthcare to Veterans who are experiencing staffing shortages at their clinic site, especially in rural areas or those with lower socioeconomic levels. For policymakers, it is vital to understand whether early implementation of this telehealth intervention provides primary care that is associated with similar quality to traditional face-to-face care. We conducted a retrospective longitudinal cohort study with two main aims: (1) identify whether chronic disease care quality measures at clinic sites that use CRH services are similar to sites that did not use CRH, and (2) examine whether these quality measures are similar between clinic sites that have used CRH services and those that have not, for sites that serve a high proportion of minority Veterans.

## METHODS

### Overview

This is a quasi-experimental analysis of data collected from a retrospective cohort of VHA clinic sites that implemented and used CRH primary care services between October 2019, when the program was rolled out nationally, and September 2021, the end of the period of early implementation. The evaluation efforts are part of an ongoing quality improvement effort at the VHA and are not considered research activity as determined by the Office of Primary Care; thus, they are not subject to institutional review board review or waiver. This study follows the Strengthening the Reporting of Observational Studies in Epidemiology (STROBE) reporting guidelines for observational studies, as well as the Standards for Quality Improvement Reporting Excellence (SQUIRE) reporting guidelines for quality improvement projects.

### Data Source(s)

The data for this study was obtained from VHA’s Corporate Data Warehouse (CDW), a national repository of clinical and administrative data from VHA’s electronic health record (EHR) system. In addition, operational reports from the National CRH Program Office were used to identify CRH-utilizing clinics, as well as their implementation and coverage dates. Electronic quality measure (eQM) data was extracted from October 2017 to September 2021 in order to observe trends both pre- and post-CRH implementation.

### Study Sample

To identify a cohort of CRH-utilizing clinics that have sufficiently implemented CRH to have a theoretical impact on primary care clinical quality measures, we defined a CRH penetration rate to determine an engagement threshold. This penetration rate is defined as the number of Veterans at a clinic with at least one CRH primary care visit divided by the number of Veterans at that clinic who are currently enrolled in primary care. We then defined a CRH-utilizing clinic as one that met the engagement threshold if the clinic had a CRH penetration rate greater than 0% for at least five out of the eight quarters in our evaluation period (FY2020–FY2021). We further required an individual clinic to meet an activity threshold of at least nine CRH primary care visits for two consecutive months. Lastly, because very small clinic sites may be differentially impacted by CRH, we limited sites to those that had at least 450 unique Veterans enrolled in primary care. The average CRH penetration rate ranged from 0.12 to 45.6%, with a median of 2.53% in the sample selected for propensity matching.

To identify a potential group of comparator clinics, we selected all clinics that did not use CRH services (CRH penetration of 0% for the entire observation period). In total, 1108 VHA primary care clinics formed the initial cohort, after excluding 243 clinics for insufficient quarterly CRH use, 865 clinics remained. A further 30 clinics had some CRH use but did not meet the threshold for consecutive month use and were excluded (*n* = 835). Finally, 132 clinics were excluded for being very small or having missing data that would prevent quality propensity matching. In total, 703 clinics were considered for matching. The propensity-matched cohort of CRH-utilizing clinics and their comparators was created by using a 1:1 match without replacement. The specific match strategy employed was optimal matching. Clinics were matched on a number of characteristics, including clinic size (as of September 2019), staffing gap measure^18^before CRH engagement (3-month average prior to national CRH start in October 2019), proportion of rural Veterans served (as of September 2019), and three access measures (new patient wait time, established patient wait time, and third next available—all 3-month averages prior to national CRH start)^19,20^. Additionally, we exact matched clinics on administrative region and whether the clinic was a VA medical center or community-based outpatient clinic (i.e., facility type). Covariates were drawn from administrative databases, including geographical data based on Rural-Urban Commuting Area (RUCA) codes. The final cohort consisted of 107 matched pairs of CRH and non-CRH-utilizing clinics (Supplemental Table 1). The statistics evaluating the quality of the match were good, with an overall absolute standardized mean difference of <0.10^21^. Table 1 outlines the characteristics of the matched cohort post propensity match (Supplemental Table 3 includes pre propensity match details).

**Table 1:** Matching Characteristics of Non-CRH-Utilizing and CRH-Utilizing Clinics Post Propensity Match

	Non-CRH-utilizing clinic, *N* = 107 (Mean (SD)/*N* (%))	CRH-utilizing clinic, *N* = 107 (Mean (SD)/*N* (%))	Standardized mean difference	95% CI	*p*-value
PC staffing gap	1.28 (0.47)	1.25 (0.29)	0.08	−0.19, 0.35	0.9
Clinic size	6879 (7153)	8178 (6824)	−0.19	−0.46, 0.08	**0.026**
Facility type					
VAMC	18 (17%)	18 (17%)	0.00	−0.27, 0.27	>0.9
CBOC/Other	89 (83%)	89 (83%)			
Proportion of rural Veterans	0.52 (0.34)	0.48 (0.35)	0.13	−0.14,0.40	0.2
Average established PC wait time	56.64 (26.10)	60.22 (24.90)	−0.13	−0.40, 0.14	0.3
Average new PC wait time	18.98 (9.60)	20.66 (11.07)	−0.16	−0.43, 0.11	0.3
Average third next available	12.87 (22.22)	13.25 (8.39)	−0.02	−0.29, 0.25	0.005
Administrative region^#^					
1	3 (2.8)	3 (2.8)	0.00	−0.27, 0.27	>0.9
2	4 (3.7)	4 (3.7)			
4	11 (10.0)	11 (10.0)			
5	2 (1.9)	2 (1.9)			
6	6 (5.6)	6 (5.6)			
7	6 (5.6)	6 (5.6)			
8	5 (4.7)	5 (4.7)			
9	0 (0.0)	0 (0.0)			
10	7 (6.5)	7 (6.5)			
12	3 (2.8)	3 (2.8)			
15	3 (2.8)	3 (2.8)			
16	9 (8.4)	9 (8.4)			
17	8 (7.5)	8 (7.5)			
19	10 (9.3)	10 (9.3)			
20	6 (5.6)	6 (5.6)			
21	9 (8.4)	9 (8.4			
22	11 (10.0)	11 (10.0)			
23	4 (3.7)	4 (3.7)			

No missing data from matched analysis

*VAMC*, VA medical center; *CBOC*, community-based outpatient clinic; *PC*, primary care

^#^Veterans’ health care is separated geographically into 18 administrative regions called Veterans Integrated Service Networks (VISNs). Each VISN is a network of medical centers and clinics that serve that region’s Veterans

### Outcome Measures

The VA tracks performance Centers for Medicare and Medicaid Services’ (CMS) Healthcare Effectiveness Data and information Set (HEDIS) based quality measures using the Electronic Quality Measurement (eQM) platform^22^. The specific outcome measures that we selected were based on results from the recent VA and RAND co-sponsored “Expert Panel on Primary Care Productivity Measurement.”^23^ This panel agreed on 12 eQMs that cover high-priority primary care conditions, have strong associations with patient outcomes, and are well established inside and outside of VHA as being core measures of primary care clinical quality. For our final analysis, nine measures were chosen (Supplemental Table 2), one measure was excluded as it was discontinued, and two depression measures were not specifically evaluated as CRH also provides specialty visits for mental health, and these will be evaluated as part of that evaluation.

Supplemental Table 2 outlines the quality measurements used in the study. The denominator of each measure was the number of all Veterans aged 18–75 years old with a documented diagnosis of diabetes, hypertension, and cardiovascular disease, respectively. The numerators were the number of Veterans who met the individual criteria for that specific measure. For example, for the proportion of patients with diabetes with poor control, the numerator was the number of Veterans diagnosed with diabetes mellitus between the ages of 18 and 75 whose HbA1c values were greater than 9 or who had no evidence of having their HbA1c measured in the prior year. The denominator was all Veterans aged 18–75 years old with a documented diagnosis of diabetes mellitus.

### Statistical Analysis

We used a comparative interrupted time series (CITS) approach to identify the causal impact of CRH on clinical quality metrics. Although quality measures are discussed en bloc, each eQM was evaluated and reported individually. The CITS approach evaluates whether the exposed group deviates from its baseline trend by a greater amount than the unexposed control group. CITS models used baseline linear trends with a random effect for clinic and adjusted for covariates that had residual imbalances post propensity score matching (clinic size, average third next available). The timeframe for our analysis was 2 years pre-intervention (October 2017–September 2019) through 2 years post-intervention (October 2019–September 2021). Outcomes are presented as the difference in the percentage of Veterans meeting the criteria for the corresponding quality measure in the post CRH period between CRH clinics and non-CRH clinics. Standard errors from all regression models were heteroskedastic robust. Missing data values for all covariate measures were excluded from the analysis prior to propensity matching. All statistical analyses were performed using R version 4.1.1. Specific packages used include MatchIt^24^and lme4^25^. A threshold of *p* < 0.05 was used to assess statistical significance.

We performed a sub-analysis where we limited the analysis to only matched clinics from the main analysis that serve a high-minority population, as we wanted to assess the potential differential impact of CRH on minority Veterans. Percentage minority serving was defined by the 25^th^ and 75^th^percentiles to categorize clinics into low, medium, and high groups^26^. Low clinics were defined as caring for <7.0% minority Veterans, medium clinics as 7.0–35.9%, and high clinics were defined as caring for >35.9% minority Veterans. The analysis was limited to only those clinics that fell into the high category (>35.9% minority). Minority status was determined based on administrative race and ethnicity records, and any Veterans who were not recorded as non-Hispanic White were considered minority. Forty-nine clinics were ultimately included in the sub-analysis.

## RESULTS

### Association of CRH on Diabetes eQMs

**Figure 1:**
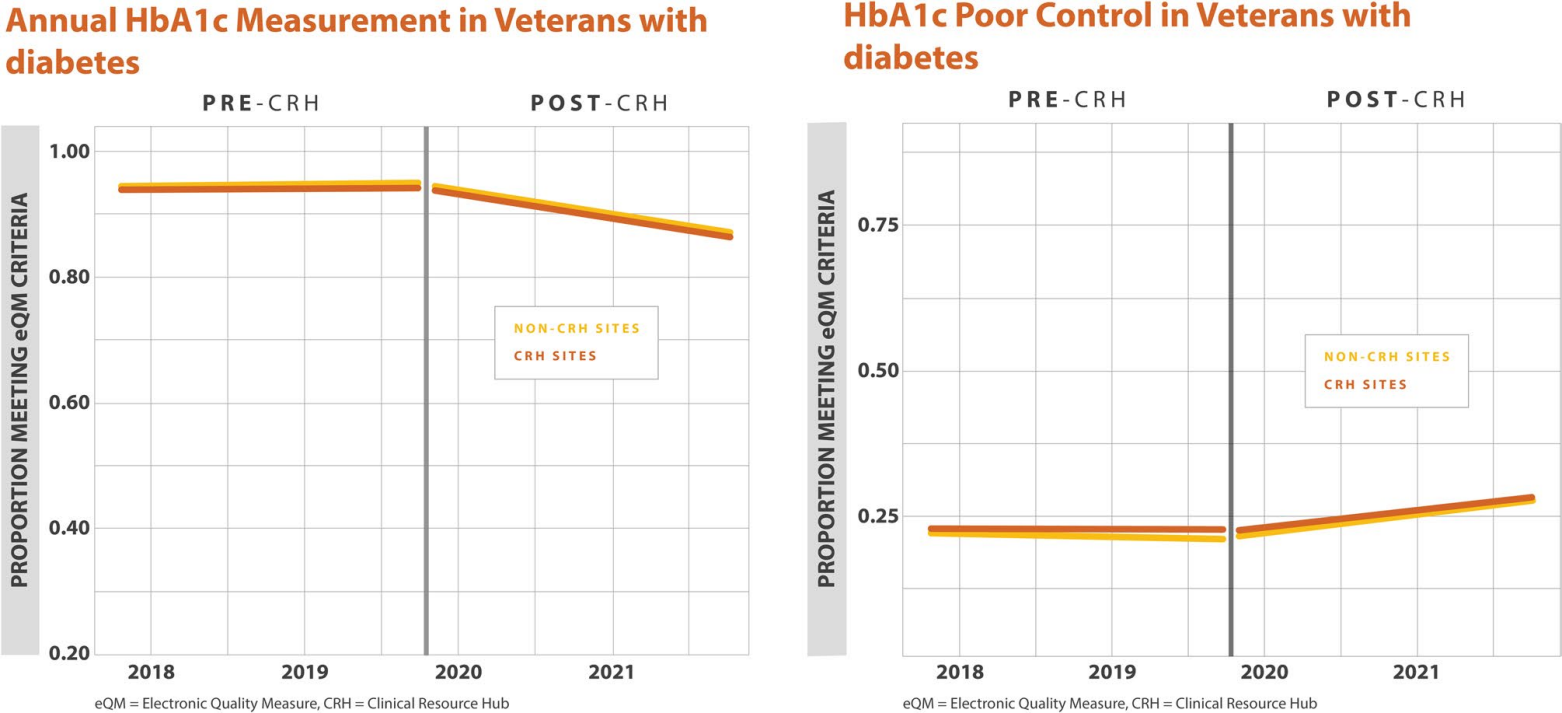
Adjusted monthly trends in electronic quality measures for diabetes, annual HbA1c measurement, and HbA1c poor control.

**Figure 2:**
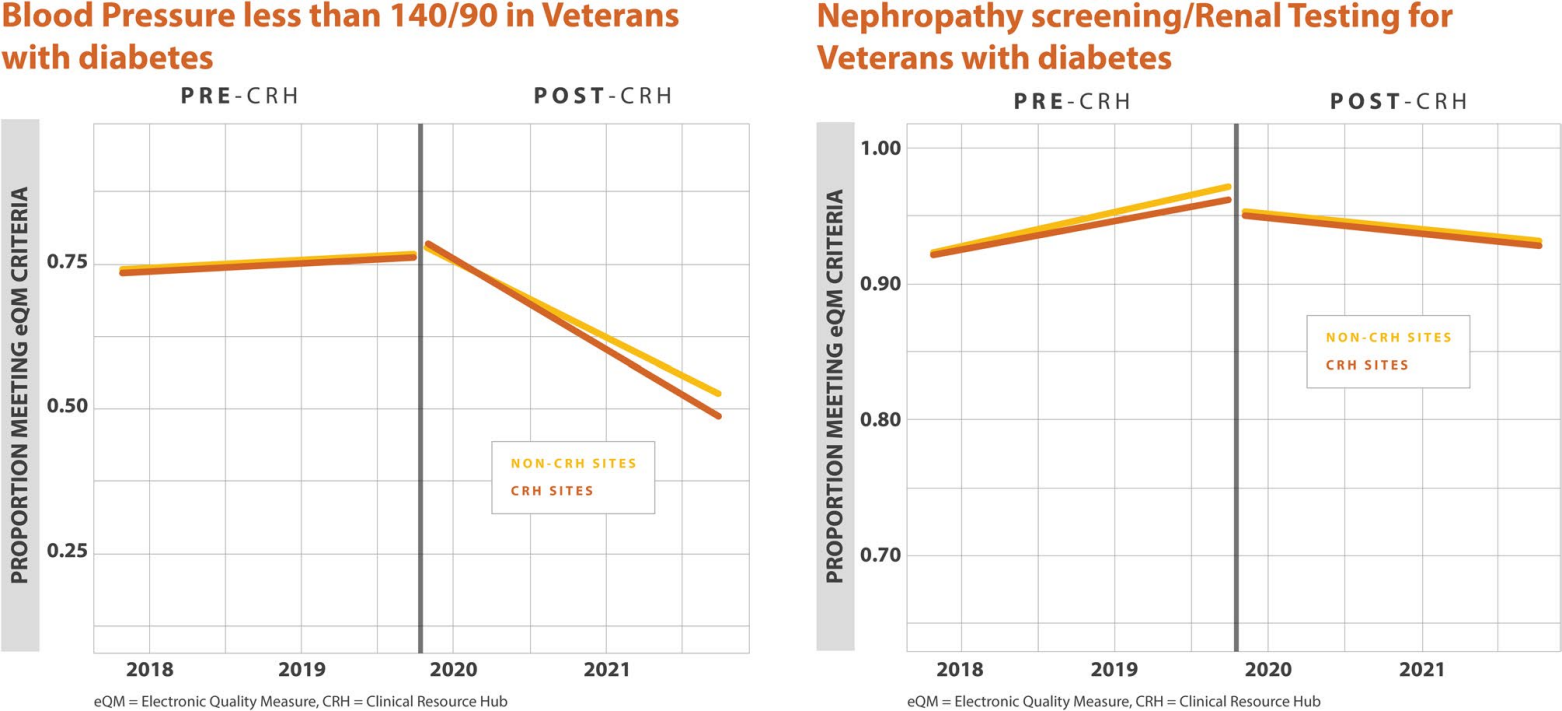
Adjusted monthly trends in electronic quality measures for diabetes, blood pressure control, and nephropathy screening.

**Figure 3:**
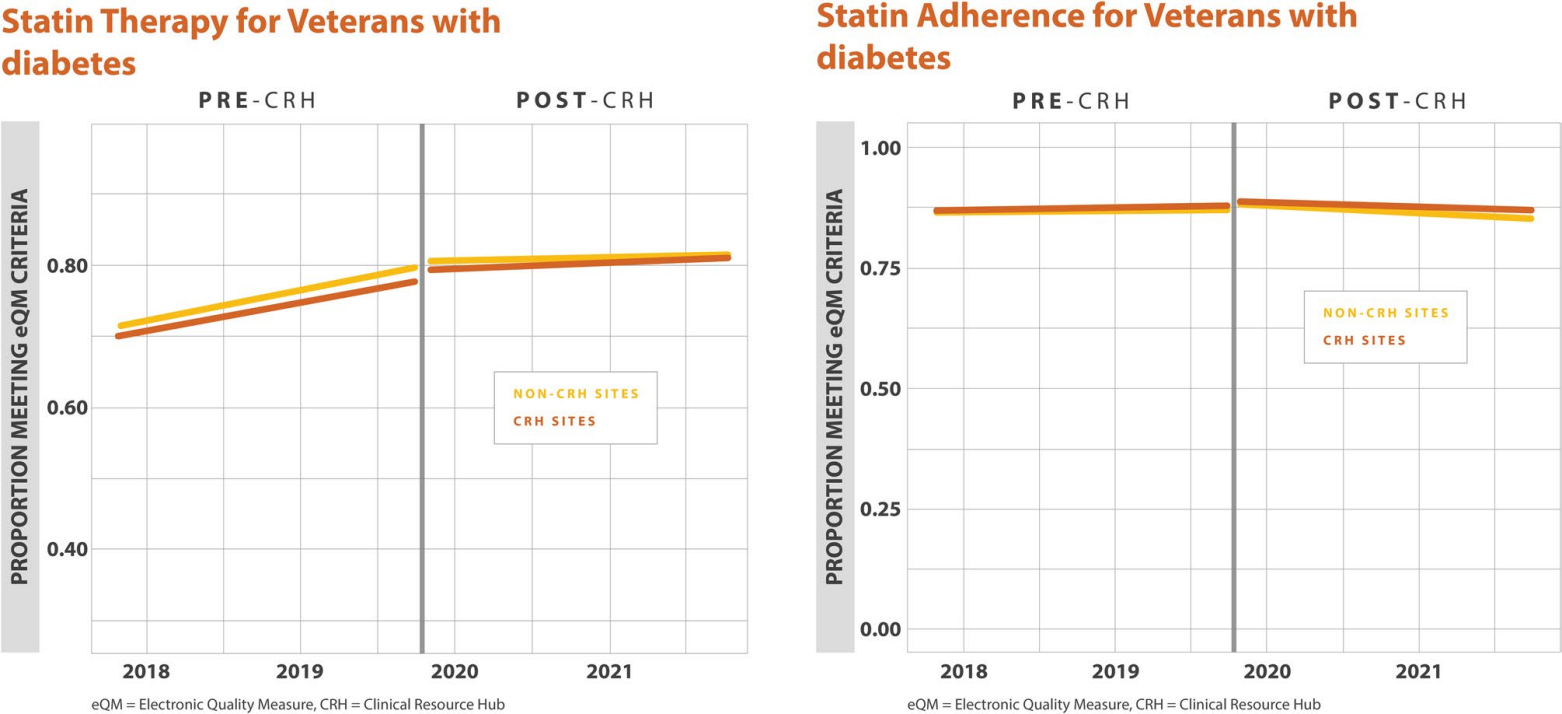
Adjusted monthly trends in electronic quality measures for diabetes, statin therapy, and statin adherence.

For annual HbA1c screening, there were no differences in the percentage of Veterans with annual HbA1c screening found between CRH-utilizing clinics and their matched controls (0.0% (–1.0%, 1.0%), *p*=0.640). For poor HbA1c control, there were no differences found between CRH-utilizing clinics and their matched controls (–1.0% (–1.0%, 0.0%), *p*=0.111) (Table 2). For blood pressure control among Veterans with diabetes, defined as a blood pressure < 140/90 mmHg, there were no differences found between CRH-utilizing clinics and their matched controls (1.0% (–0.0%, 3.0%),* p*=0.095). We found a higher percentage of Veterans with diabetes and nephropathy screening at CRH-utilizing clinics compared to non-CRH-utilizing clinics (1.0% (0.0%, 1.0%), *p* =0.010). For statin therapy for Veterans with diabetes, we found a higher percentage of Veterans meeting this measure at CRH-utilizing clinics compared to non-CRH-utilizing clinics (1.0% (0.0%,1.0%), *p*=0.003). For statin adherence for Veterans with diabetes, there were no differences found between CRH-utilizing clinics and non-CRH-utilizing clinics (0.0% (–1.0%,0.0%), *p*=0.292) (Table 2, Figs. 1, 2, 3).

**Table 2:** Percentage Differences Between CRH-Utilizing Clinics and Non-CRH-Utilizing Clinics for Quality Measures

Disease group	Measure	Main analysis		Minority sub-analysis	
		Percentage difference (95% CI)	*p*-value	Percentage difference (95% CI)	*p*-value
Diabetes	Annual HbA1c Measurement in Veterans with diabetes	0.0% (−1.0%, 1.0%)	0.640	1.0% (0.0%, 2.0%)	0.044
Diabetes	HbA1c poor control in Veterans with diabetes	−1.0% (−1.0%, 0.0%)	0.111	−1.0% (−2.0%, 0.0%)	0.236
Diabetes	BP less than 140/90 in Veterans with Diabetes	1.0% (−0.0%, 3.0%)	0.095	0.0% (−3.0%, 3.0%)	0.915
Diabetes	Nephropathy screening/Renal Testing for Veterans with diabetes	1.0% (0.0%, 1.0%)	0.010	1.0% (0.0%, 2.0%)	0.023
Diabetes	Statin therapy for Veterans with diabetes	1.0% (0.0%, 1.0%)	0.003	1.0% (0.0%, 2.0%)	0.007
Diabetes	Statin adherence for Veterans with diabetes	0.0% (−1.0%, 0.0%)	0.292	−1.0% (−3.0%, 0.0%)	0.015
Hypertension	Controlling High Blood Pressure in Veterans with hypertension	1.0% (−1.0%, 2.0%)	0.382	1.0% (−2.0%, 3.0%)	0.493
Cardiovascular disease	Statin therapy for Veterans with cardiovascular disease	0.0% (−1.0%, 2.0%)	0.348	0.0% (−2.0%, 2.0%)	0.945
Cardiovascular disease	Statin adherence for Veterans with cardiovascular disease	−1.0% (−3.0%, 1.0%)	0.467	1.0% (−2.0%, 5.0%)	0.490

Although eQMs are discussed en bloc, estimates in this table are reported individually

*HbA1c*, hemoglobin A1c; *BP*, blood pressure

### Association of CRH on Hypertension eQMs

**Figure 4:**
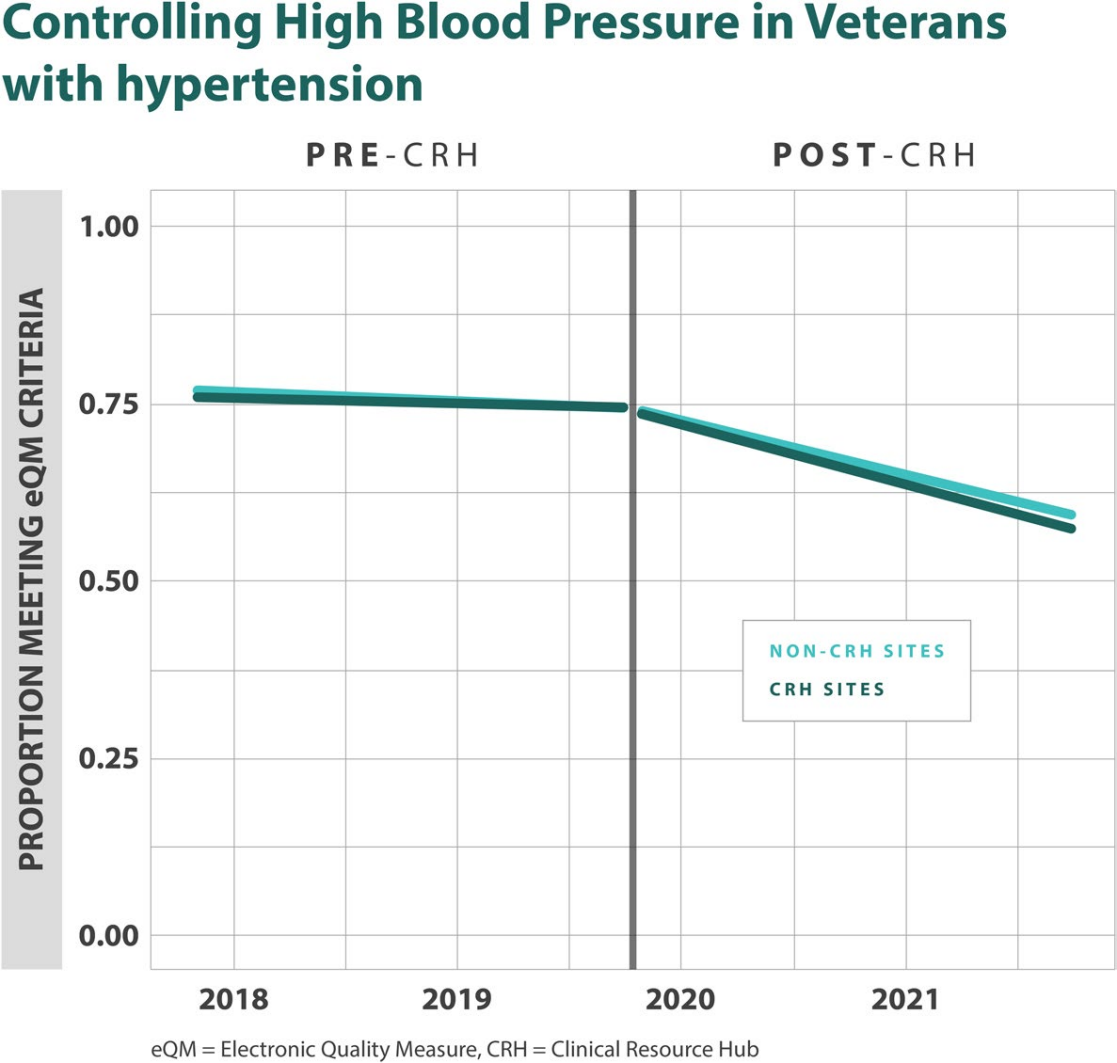
Adjusted monthly trends in electronic quality measures for hypertension, blood pressure control.

We did not find a statistically significant difference in the control of blood pressure when CRH-utilizing clinics were compared to their matched controls (1.0% (–1.0%,2.0%), *p*=0.382) (Table 2, Fig. 4).

### Association of CRH on Cardiovascular Disease eQMs

**Figure 5:**
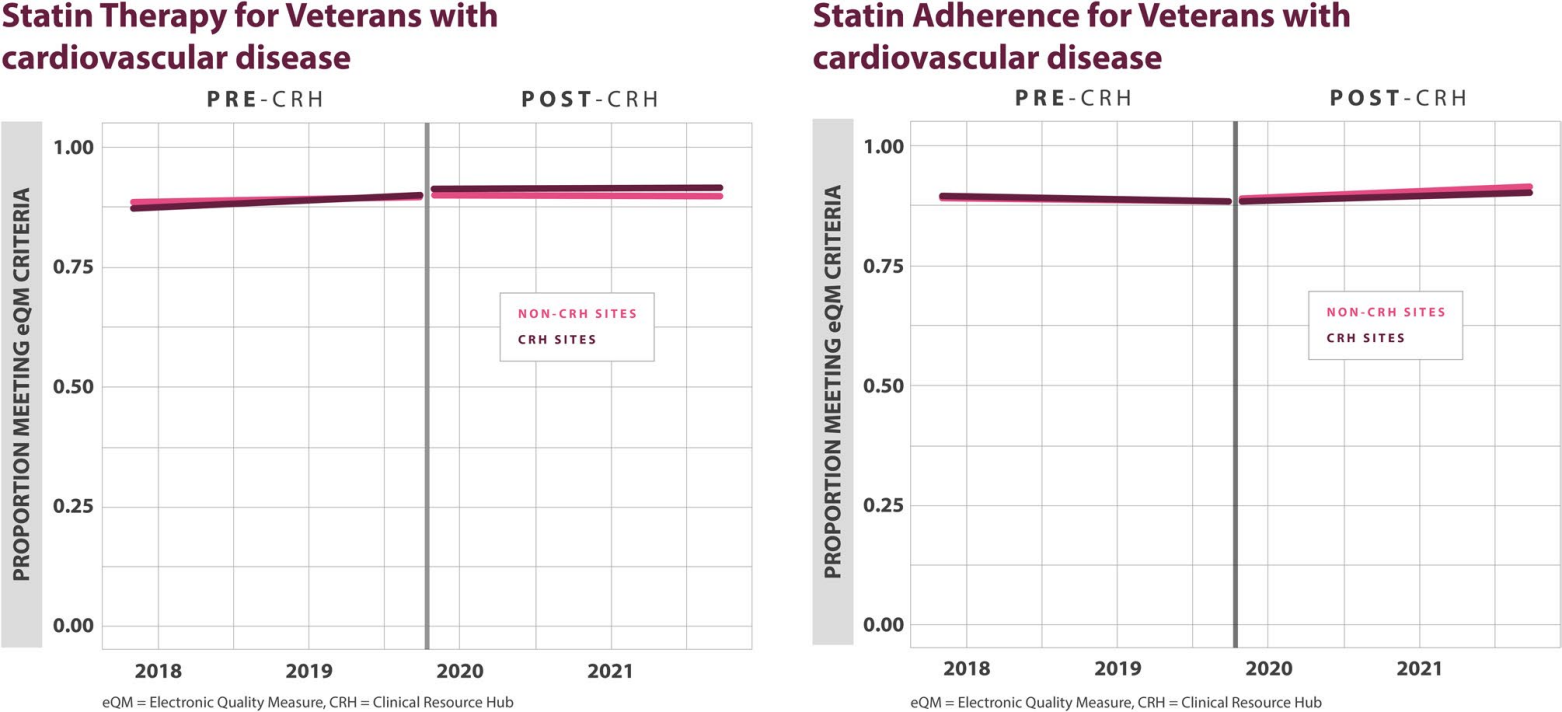
Adjusted monthly trends in electronic quality measures for cardiovascular disease, statin therapy, and statin adherence.

For statin therapy for Veterans with cardiovascular disease, there were no differences found between CRH-utilizing clinics and non-CRH-utilizing clinics (0.0% (–1.0%, 2.0%), *p*=0.348). For statin adherence or Veterans with cardiovascular disease, there were no differences found between CRH-utilizing clinics and non-CRH-utilizing clinics (–1.0% (–3.0%, 1.0%), *p*=0.467) (Table 2, Fig. 5).

### Sub-analysis Impact on Clinics that Serve a High Proportion of Minority Veterans

For annual HbA1c screening and when limiting the analysis to only clinics that serve a high proportion of minority Veterans, defined as >35.9% minority, we found a higher percentage of Veterans meeting this measure at CRH-utilizing clinics compared to non-CRH-utilizing clinics (1.0% (0.0%, 2.0%), *p*=0.044). For poor HbA1c control, there were no differences found between CRH-utilizing clinics and their matched controls (–1.0% (–2.0%, 0.0%), *p*=0.236). For blood pressure control among Veterans with diabetes, defined as a blood pressure < 140/90 mmHg, there were no differences found between CRH-utilizing clinics and their matched controls (0.0% (–3.0%, 3.0%), *p*=0.915). We found a higher percentage of Veterans with diabetes and nephropathy screening at CRH-utilizing clinics compared to non-CRH-utilizing clinics (1.0% (0.0%, 2.0%), *p* =0.023). For statin therapy for Veterans with diabetes, we found a higher percentage of Veterans meeting this measure at CRH-utilizing clinics compared to non-CRH-utilizing clinics (1.0% (0.0%, 2.0%), *p*=0.007). For statin adherence for Veterans with diabetes, there was a lower percentage of Veterans meeting this measure at CRH-utilizing clinics compared to non-CRH-utilizing clinics (–1.0% (–3.0%,0.0%), *p*=0.015).

There were no statistically significant differences between CRH-utilizing clinics and their matched comparator clinics for blood pressure control when limiting the sample to only clinics that care for a high percent of minority Veterans (1.0% (–2.0%, 3.0%),* p*=0.493).

For statin therapy for Veterans with cardiovascular disease and when limiting the analysis to only clinics that serve a high proportion of minority Veterans, there were no differences found between CRH-utilizing clinics and non-CRH-utilizing clinics (0.0% (–2.0, 2.0%), *p*=0.954). For statin adherence for Veterans with cardiovascular disease, there were no differences found between CRH-utilizing clinics and non-CRH-utilizing clinics (1.0% (–2.0%, 5.0%), *p*=0.490) (Table 2).

## DISCUSSION

In this quality improvement study of the Veteran Health Administration’s national implementation of the Clinical Resource Hub telehealth initiative, we found that primary care quality measures at CRH-utilizing clinics undergoing early implementation of the program were similar to those of matched comparator clinics that have not implemented CRH. Furthermore, in the majority of cases, there were no differences in quality outcomes among clinics that serve a high level of minority Veterans.

Our findings can best be understood in the context of understanding the key functions of primary care^27–31^, including access, continuity, coordination, and comprehensiveness. In the context of Veterans receipt of CRH services, access and continuity may be the most relevant. CRH likely improves access by providing needed care to Veterans who have lost access to primary care services. However, CRH care likely is disruptive to interpersonal continuity as the Veteran is seeing a new provider. While this care may be provided by a new provider, there is consistent informational continuity in VHA due to VHA’s investment in health information technology. Health information technology makes it easier for providers to access information about a Veteran’s condition, medications, previous treatments, and other relevant information, thus providing informational continuity. Prior work has shown that loss of an individual provider may not have a significant impact on chronic and preventive care quality if information continuity is maintained^32^. Previously published literature has shown that patients using telehealth had similar or better performance measurement scores. Common medical conditions managed in primary care, such as diabetes, hypertension, and cardiovascular disease, are of special interest in determining whether telehealth is associated with similar quality metrics to face-to-face care due to their corresponding significant morbidity and mortality^32–35^.

We did not find any evidence that there were differences in the findings at clinics serving a high percentage of minority Veterans^32^. There is evidence in the literature that telehealth interventions are less likely to be used by patients with limited English proficiency and for racial and ethnic minorities^15–17,36^, often as a result of lower digital literacy and/or internet access compared to non-Hispanic White individuals. Interventions that are technology-based are especially prone to “intervention-generated” inequities and can result from differential access, uptake, adherence, and/or effectiveness^37,38^. The CRH program is specifically mandated to serve Veterans in at-risk areas for disparities, such as rural and medically underserved areas. It is possible that this is the reason why our results differ from the results of evaluations of other telehealth interventions.

### Limitations

We acknowledge several limitations to this evaluation. First, this is an observational study that can only show associations, not causation. However, the comparative interrupted time series (CITS) approach is a rigorous quasi-experimental research design that allows us to control for many sources of bias. In addition, one of the primary limitations of the CITS approach is when it is limited to a small number of evaluation time points pre and post intervention. In the case of this evaluation, we observed 24 separate time points pre-intervention and 24 time points post-intervention, giving more credibility to our findings. Second, this analysis was done at a clinic level, so we are unable to draw inference on individual quality metrics at a Veteran level; however, CRH was proposed as a clinic-level intervention.

Third, it is possible for quality metrics to be completed outside the VA, resulting in a missed quality measure for an individual Veteran. Fourth, there are likely many co-occurring interventions happening at primary care clinics that may impact clinical quality outcomes outside of CRH. Our use of a matched cohort of clinics within similar regions can overcome some of these limitations, as CRH-utilizing and non-CRH-utilizing clinics should have similar exposure to other interventions. Lastly, the average CRH penetration rate varied widely over the sample of sites for this analysis, and future work should examine how differing levels of CRH impact quality outcomes.

## CONCLUSION

Primary care quality was either similar or slightly better for the CRH program utilizing clinics compared to comparable non-CRH-utilizing clinics during the period of early implementation of VHA’s national CRH program. In addition, there was no evidence that the CRH program contributed to differences in primary care quality at clinics serving a high percentage of minority Veterans. These findings have important implications for the VHA. First, these results provide early support that telehealth interventions can improve access to primary health care in a variety of settings, especially in clinics that are experiencing staff shortages, without impacting quality of care. Second, it is reassuring that our findings did not show any evidence of exacerbating existing racial and/or ethnic disparities which have historically been associated with telehealth. Future work should focus on other potential disparities such as age and lower digital literacy to ensure that CRH is not inducing disparities in other areas. In addition, future evaluation of individual Veteran-level characteristics will complement these site-level findings. Lastly, these results can have important implications for other large integrated healthcare systems that have implemented or are in the process of implementing telehealth programs in primary care.

## Supplementary Information

Below is the link to the electronic supplementary material.

Supplementary file1 (DOCX 28 KB)

Supplementary file2 (DOCX 15 KB)

Supplementary file3 (DOCX 21 KB)

## Data Availability

All raw data are the property of the United States Government; data availability will be subject to review by the Department of Veterans Affairs and must be in compliance with all applicable federal policies and laws.
