# Supplementary material for: Early Implementation of a Regional Telehealth Contingency Staffing Program and Primary Care Quality in the Veterans Health Administration: Evidence from the Clinical Resource Hub program
Source: J Gen Intern Med. 2025 May 20;40(14):3353–62. doi: 10.1007/s11606-025-09615-2 (PMC12586748; doi:10.1007/s11606-025-09615-2)
Supplement: Supplementary file 1 — Supplementary file1 (DOCX 28 KB) [file 11606_2025_9615_MOESM1_ESM.docx]

243 clinics excluded for insufficient quarterly CRH use

132 clinics excluded for being very small or missing data

30 clinics excluded for insufficient consecutive month CRH use

**214 Final Sample**

(107 non-CRH utilizing;

107 CRH utilizing)

**703 Primary Care Clinics Considered for Matching**

(557 non-CRH utilizing;

146 CRH utilizing)

**835 Primary Care Clinics**

(686 non-CRH utilizing;

149 CRH utilizing)

**865 Primary Care Clinics**

(686 non-CRH utilizing;

179 CRH utilizing)

**1108 Primary Care Clinics**
