# Supplementary material for: Early Implementation of a Regional Telehealth Contingency Staffing Program and Primary Care Quality in the Veterans Health Administration: Evidence from the Clinical Resource Hub program
Source: J Gen Intern Med. 2025 May 20;40(14):3353–62. doi: 10.1007/s11606-025-09615-2 (PMC12586748; doi:10.1007/s11606-025-09615-2)
Supplement: Supplementary file 2 — Supplementary file2 (DOCX 15 KB) [file 11606_2025_9615_MOESM2_ESM.docx]

**Supplemental Table 2**: Primary care quality measures: Chronic disease management measures (eQMs)

| *Measure* |  |  |  | *Description* | *Numerator* | *Denominator* | *Preferred Score Direction* |
| --- | --- | --- | --- | --- | --- | --- | --- |
| c9h_ec |  |  |  | Annual HbA1c Measurement in Veterans with diabetes | Veterans with documentation of annual measurement of HbA1c | Veterans 18-75 years old with a diagnosis of DM | Higher is better |
| dmg23h_ec |  |  |  | HbA1c poor control in Veterans with diabetes | Veterans with most recent HbA1c greater than 9 or no evidence of test within measurement year | Veterans 18-75 years old with a diagnosis of DM | Lower is better |
| dmg27h_ec |  |  |  | BP less than 140/90 in Veterans with Diabetes | Veterans with most recent recorded BP as < 140/90mmHg | Veterans 18-75 years old with a diagnosis of DM | Higher is better |
| dmg34h_ec |  |  |  | Nephropathy screening/Renal Testing for Veterans with diabetes | Veterans with documented screening for nephropathy within measurement year | Veterans 18-75 years old with a diagnosis of DM | Higher is better |
| statn7_ec |  |  |  | Statin therapy for Veterans with diabetes | Veterans with at least one dispensing of statin of any intensity within measurement year | Veterans 40-75 years old with a diagnosis of DM | Higher is better |
| statn8_ec |  |  |  | Statin adherence for Veterans with diabetes | Veterans who have a statin prescribed for 80% of treatment period within measurement year | Veterans 40-75 years old with a diagnosis of DM | Higher is better |
| ihd53h_ec |  |  |  | Controlling High Blood Pressure in Veterans with hypertension | Veterans with most recent recorded BP as < 140/90mmHg | Veterans 18-85 years old with a diagnosis of hypertension | Higher is better |
| statn1_ec |  |  |  | Statin Therapy for Veterans with cardiovascular disease | Veterans with at least one dispensing event of high/moderate statin within measurement year | Male Veterans 21-75 years old/Female Veterans 40-75 with a diagnosis of CVD | Higher is better |
| statn4_ec |  |  |  | Statin adherence for Veterans with cardiovascular disease | Veterans who have a high/moderate statin for 80% of the treatment period within measurement year | Male Veterans 21-75 years old/Female Veterans 40-75 with a diagnosis of CVD | Higher is better |

*Abbreviations: HbA1c = Hemoglobin A1c; BP = Blood pressure; DM = diabetes mellitus*
