# Supplementary material for: Early Implementation of a Regional Telehealth Contingency Staffing Program and Primary Care Quality in the Veterans Health Administration: Evidence from the Clinical Resource Hub program
Source: J Gen Intern Med. 2025 May 20;40(14):3353–62. doi: 10.1007/s11606-025-09615-2 (PMC12586748; doi:10.1007/s11606-025-09615-2)
Supplement: Supplementary file 3 — Supplementary file3 (DOCX 21 KB) [file 11606_2025_9615_MOESM3_ESM.docx]

**Supplemental Table 2:** Pre-propensity matching characteristics of all VA primary care clinics

|  |  | **All Primary Care Clinics, N = 1,108**  **(Mean (SD)/N (%))** | **Non-CRH utilizing clinic,**  **N = 686**  **(Mean (SD)/N (%))** | **CRH utilizing clinic,**  **N = 179**  **(Mean (SD)/N (%))** | **Standardized Mean Difference** | **95% CI** | **p-value** |
| --- | --- | --- | --- | --- | --- | --- | --- |
| **PC staffing gap**  *Missing data 6.0%* |  | 3.13 (41.3) | 4.29 (53.1) | 1.35 (0.64) | 0.08 | -0.09, 0.24 | 0.2 |
| **Clinic size**  *Missing data 4.6%* |  | 5,924 (6,504) | 4,046 (4,963) | 10,401 (7,659) | -0.99 | -1.2, -0.81 | **<0.001** |
| **Facility type**  *Missing data 0.6%* |  |  |  |  | 0.97 | 0.80, 1.1 | **<0.001** |
| VAMC |  | 169 (15%) | 32 (4.7%) | 74 (41%) |  |  |  |
| CBOC/Other |  | 934 (85%) | 649 (95%) | 105 (59%) |  |  |  |
| **Proportion of rural Veterans**  *Missing data 4.7%* |  | 0.52 (0.36) | 0.56 (0.37) | 0.45 (0.33) | 0.32 | 0.15, 0.49 | **<0.001** |
| **Average established PC wait time**  *Missing data 6.0%* |  | 56.06 (29.33) | 56.09 (30.60) | 55.31 (25.29) | 0.03 | -0.14, 0.19 | >0.9 |
| **Average new PC wait time**  *Missing data 10.0%* |  | 17.43 (9.42) | 16.45 (9.22) | 19.58 (9.92) | -0.33 | -0.50, -0.16 | **<0.001** |
| **Average third next available**  *Missing data 6.5%* |  | 10.47 (10.44) | 9.50 (11.73) | 12.72 (7.72) | -0.33 | -0.49, -0.16 | **<0.001** |
| **Administrative Region^#^**  *Missing data 0.0%* |  |  |  |  | 0.76 | 0.60, 0.93 | **<0.001** |
| 1 |  | 54 (4.9%) | 39 (5.7) | 3 (2.8) |  |  |  |
| 2 |  | 73 (6.6%) | 56 (8.2) | 8 (3.7) |  |  |  |
| 4 |  | 55 (5.0%) | 31 (4.5) | 15 (10.0) |  |  |  |
| 5 |  | 38 (3.4%) | 23 (3.4) | 4 (1.9) |  |  |  |
| 6 |  | 43 (3.9%) | 23 (3.4) | 6 (5.6) |  |  |  |
| 7 |  | 66 (6.0%) | 48 (7.0) | 6 (5.6) |  |  |  |
| 8 |  | 71 (6.4%) | 43 (6.3) | 8 (4.7) |  |  |  |
| 9 |  | 50 (4.5%) | 42 (6.1) | 2 (0.0) |  |  |  |
| 10 |  | 74 (6.7%) | 37 (5.4) | 16 (6.5) |  |  |  |

|  |  | **All Primary Care Clinics, N = 1,108**  **(Mean (SD)/N (%))** | **Non-CRH utilizing clinic,**  **N = 686**  **(Mean (SD)/N (%))** | **CRH utilizing clinic,**  **N = 179**  **(Mean (SD)/N (%))** | **Standardized Mean Difference** | **95% CI** | **p-value** |
| --- | --- | --- | --- | --- | --- | --- | --- |

^#^*Veterans’ health care is separated geographically into 18 administrative regions called Veterans Integrated Service Networks (VISNs). Each VISN is a network of medical centers and clinics that serve that region’s Veterans.*

| 12 |  | 48 (4.3%) | 32 (4.7) | 5 (2.8) |  |  |  |
| --- | --- | --- | --- | --- | --- | --- | --- |
| 15 |  | 71 (6.4%) | 61 (8.9) | 5 (2.8) |  |  |  |
| 16 |  | 61 (5.5%) | 36 (5.2) | 13 (8.4) |  |  |  |
| 17 |  | 58 (5.2%) | 38 (5.5) | 10 (7.5) |  |  |  |
| 19 |  | 84 (7.6%) | 50 (7.3) | 16 (9.3) |  |  |  |
| 20 |  | 52 (4.7%) | 10 (1.5) | 24 (5.6) |  |  |  |
| 21 |  | 59 (5.3%) | 30 (4.4) | 11 (8.4) |  |  |  |
| 22 |  | 77 (6.9%) | 45 (6.6) | 18 (10.0) |  |  |  |
| 23 |  | 74 (6.7%) | 42 (6.1) | 9 (3.7) |  |  |  |
